# Dynamical system of a time-delayed *ϕ*^6^-Van der Pol oscillator: a non-perturbative approach

**DOI:** 10.1038/s41598-023-38679-5

**Published:** 2023-07-24

**Authors:** Galal M. Moatimid, T. S. Amer

**Affiliations:** 1 Department of Mathematics, Faculty of Education, Ain Shams University, Cairo, Egypt; 2 Department of Mathematics, Faculty of Science, Tanta University, Tanta, 31527 Egypt

**Keywords:** Mathematics and computing, Applied mathematics

## Abstract

A remarkable example of how to quantitatively explain the nonlinear performance of many phenomena in physics and engineering is the Van der Pol oscillator. Therefore, the current paper examines the stability analysis of the dynamics of *ϕ*^6^-Van der Pol oscillator (PHI6) exposed to exterior excitation in light of its motivated applications in science and engineering. The emphasis in many examinations has shifted to time-delayed technology, yet the topic of this study is still quite significant. A non-perturbative technique is employed to obtain some improvement and preparation for the system under examination. This new methodology yields an equivalent linear differential equation to the exciting nonlinear one. Applying a numerical approach, the analytical solution is validated by this approach. This novel approach seems to be impressive and promising and can be employed in various classes of nonlinear dynamical systems. In various graphs, the time histories of the obtained results, their varied zones of stability, and their polar representations are shown for a range of natural frequencies and other influencing factor values. Concerning the approximate solution, in the case of the presence/absence of time delay, the numerical approach shows excellent accuracy. It is found that as damping and natural frequency parameters increase, the solution approaches stability more quickly. Additionally, the phase plane is more positively impacted by the initial amplitude, external force, damping, and natural frequency characteristics than the other parameters. To demonstrate how the initial amplitude, natural frequency, and cubic nonlinear factors directly affect the periodicity of the resulting solution, many polar forms of the corresponding equation have been displayed. Furthermore, the stable configuration of the analogous equation is shown in the absence of the stimulated force.

## Introduction

The subsequent second-order non-autonomous ordinary differential equations (ODEs) as given in Eqs. (1) and (2), respectively, give a typical form of the Van der Pol and Duffing oscillators prototypes through excitations:

1 $$\ddot{x} - \mu (1 - x^{2} )\dot{x} + \frac{dV(x)}{{dx}} = F_{1} \cos \omega_{1} t,$$

2 $$\ddot{x} + \lambda \dot{x} + \frac{dV(x)}{{dx}} = F_{2} \cos \omega_{2} t,$$

where *x* is the state variable, dots over *x* denote derivatives with time, *μ* and *λ* are the dampening parameters, *F*_*j*_ and $\omega_{j} (j = 1,\;2)$ are the amplitude of the external excitation and angular frequency, respectively, and *V*(*x*) is a potential. The Van der Pol oscillator is regularly define the self-excited oscillators in physics, engineering, electronics, biology, neurology, and many other specialties. Alternatively, the Duffing oscillator model depicts numerous physical, electrical, mechanical, and engineering machines. The potentials *V*(*x*) are estimated by limited Taylor series for the $\phi^{2} ,\;\phi^{4} ,$ and $\phi^{6}$ chaotic oscillators as in the following Eqs. (3–5), correspondingly.

3 $$V(x) = \frac{1}{2}\alpha x^{2} ,$$

4 $$V(x) = \frac{1}{2}\alpha x^{2} + \frac{1}{4}\beta x^{4} ,$$

5 $$V(x) = \frac{1}{2}\alpha x^{2} + \frac{1}{4}\beta x^{4} + \frac{1}{6}\lambda x^{6} ,$$

where $\alpha ,\;\beta ,$ and $\lambda$ are parameters of the potentials. The classification of the previous physical parameters and further details about these equations were investigated^1^. A dynamic of PHI6 prototype response to an external excitation was studied^2^. Numerical simulations were used to show the non-autonomous oscillator potential (PPHI6) of periodic and chaotic motions. Phase diagrams revealed several steady-state types, demonstrating that variability was present for a variety of external force. The creation of the chaotic oscillator was an interesting phenomenon that has accumulated a lot of scientific attention in recent years. A significant amount of recent research on chaotic matching originated from various fascinating real-world applications, including those in network security, chaos-generating designs, chemical reactions, lasers, biological systems, information science, neural networks, etc. In a PPHI6, the dynamics of a periodically pushed particle were considered^3^. The multiple times scales technique produced harmonic, subharmonic, and super-harmonic oscillatory states. Numerical simulations that demonstrated the fractality of the attraction basins were used to supplement the findings. In cases where the PPHI6 was a bounded or unbounded double hump, the presence of homoclinic bifurcation was investigated^4^. The fractal basin boundary served as an easy way to illustrate the precision of the method. Whether multi-parameter oscillators using PPHI6 might harmonize adaptively was investigated^5^. It was demonstrated that a single-state adaptive feed-back was adequate to guide two identical oscillators to stable synchronization and was taken into consideration when considering the synchronization for known and unknown system parameters for the PHI6. Feng^6^ was interested in the first integrals of the Duffing–Van der Pol prototype that were taken into consideration under specific parametric conditions. The first integrals of the original oscillator system under the specified parametric circumstances were obtained using inverse transformations, and some special cases of these equations were provided correspondingly. A new approximation approach was proposed for the nonlinear Duffing–Van der Pol oscillator^7^. In order to solve the challenge of accurately recording the behavior of the solution and providing a good approximation to the solution for a considerable amount of time, the suggested technique offered an alternative methodology. The Duffing–Van der Pol equation yielded a traditional approximate solution in the presence of secular terms^8^. Unfortunately, it was unable to disregard these secular factors using the conventional approach. Therefore, a bounded approximation solution was attained using the extended frequency approach. In light of the significance of the previous aspects, the current work aims to examine the PHI6 structure.

Numerous engineering, physics, chemistry, biology, and economic systems all have time delays. The stability and performance of control systems were significantly impacted by the time delay caused by sensor and actuator dynamics, signal transmission, and digital computations. The fact that a time delay was frequently unknown made matters worse. It was difficult to estimate time delays in a control system. When the system dynamics were nonlinear and unknown, it became considerably more difficult. The identification of nonlinear dynamic systems and the estimation of the time delay caused by the feedback control were accomplished using a nonparametric identification technique^9^. The suggested algorithm coupled cross-validation machine learning techniques, for automatic model selection with an algebraic operation for preprocessing signals to remove the dependency on beginning conditions and filter out noise. A nonlinear Duffing oscillator was simulated to show the suggested effectiveness and precision of the method. Following a brief discussion of the driving factors behind the research of time-delay systems, modifications (models, stability, and structure) brought about by the existence of the delay phenomena were reported^10^. The sliding mode and time-delay controls in particular received a quick ramshackle of some control strategies. A simulation was used to confirm the functionality of an algorithm for the precise least-squares identification of an approximate continuous-time time-delay system^11^. A recursive approach was introduced for the online identification of systems with unpredictable time delays^12^. Both techniques were simply modifications of the least-squared approach in general. The technique was suggested to use a discrete-time model and a recursive identification method for fractional time-delay systems^13^. It offered a new framework for system identification and discussed the discretization of fractional time-delay systems. Additionally, numerical examples were used to verify the efficiency of the suggested strategy. Based on instrumental variable identification techniques, a methodology to estimate continuous-time properties including time delay was examined^14^. It made use of the helpful redundancy strategy to get over the numerous local minima of the cost function connected to the estimation of a time delay system. It investigated how to identify unknown parameters and account for time delays in dynamic systems^15^. After changing the system structure using a polynomial function, appropriate neural network methods for determining time-lag system parameters were created. It was suggested that applications to hybrid systems switches were delayed^16^. The findings of experimental studies on a delay process as well as numerical simulations using noisy data were presented. Investigations on time delay systems identifiability and algebraic identification were conducted^17^. First, identification results for linear delay systems defined by convolution equations were shown. Experimental findings and simulation experiments with noisy data demonstrated the effectiveness of the suggested technique. For the impulsive noise environment, a brand-new adaptive time delay estimation approach with amplitude attenuation was presented^18^. A closed form of the recursive solution for the direct approach, which has no free parameters unlike the two-step algorithm in gradient-based techniques, was provided to increase the strength of the proposed two-step algorithm. Time-delayed position and speed were employed to suppress the nonlinearity of an excited Van der Pol-Duffing oscillator^19–21^. The time delay served as an additional protection against the system under consideration vibrating nonlinearly. Computing was inspired to explore the behavior of multi-delay differential systems that highlighted the effect of latent time through the exploitation/exploration of artificial intelligence^22^. To examine the resolution of the nonlinear multiple delays, an original concept of a smart computer paradigm employing the strengths/knacks of based technique via two-layer structure networks was given^23^. For the numerical study of the nonlinear delay differential system for dynamics of plant virus propagation with the effect of seasonality and delays, the artificially intelligent knacks-based stochastic paradigms has been used^24^. To further explain the structure of Dengue intracellular propagation dynamics, non-integer order specimens with timing delay was investigated^25^. Shah et al.^26^ demonstrated how factors like as migration, protection, mortality rate, exposure, treatment rate, and contact between infected and healthy persons have an impact on the populace. In this model, there were four classes: susceptible, exposed, infected, and recovering. Because technologies with a temporal delay have recently been the focus of numerous investigations, the topic of this work is particularly recent.

It is well known that nonlinear ODEs could be used to construct a variety of engineering problems. Despite the fact that they have few precise responses and appear to be extremely difficult, the general solutions of these classes were impossible. Due to this circumstance, asymptotic solutions to various nonlinear equations have attracted the attention of several researchers. Examples include the small parameter technique and the averaging method for some weak nonlinear problems^27, 28^. Investigations on the dynamical characteristics of a pendulum coupled to a rigid rotating framework with a uniform angular speed about the vertical axis going through the pivot point of the pendulum were made^27^. The governing equation of motion was an analytical solution obtained using the homotopy perturbation method (HPM). The Lyapunov exponent and moment Lyapunov exponents of linear systems with two degrees of freedom and white noise parametric excitation were examined^28^. The explicit asymptotic equations for these exponents were calculated using the HPM in the presence of small-intensity sounds. The numerical Monte Carlo simulation method for this stochastic system was used to verify the accuracy of the approximations for the moment Lyapunov exponents. These approaches, however, rely on a little parameter, and poor choice of this parameter resulted in incorrect solutions^29^. Identifying the little parameter and the scale required to establish the relevant equations in a more convenient form for the practical application of the method was the key step in any asymptotic or perturbation method. In recent years, iterative approaches that use the HPM were important in producing approximations of a large variety of nonlinear problems that were reasonably close to their closed-form solutions^30^. These approaches depended on the initial solution estimate, thus if the first guess was not close to the final solution of the problem, it may diverge, which will prevent the process from achieving the intended solution. The investigation of an infinite range of vibrational frequencies was one of the challenging problems that give rise to difficulties involving nonlinear oscillators, and has been studied by numerous researchers. Due to the complexity of nonlinearity, it was challenging for scientists and applied physicists to arrive at an exact or semi-precise solution to certain nonlinear equations. Prof., He derived the frequency formula and came up with a wonderful solution by transforming a nonlinear equation roughly to a linear one^31–36^. An overview of some recent advancement in asymptotic methods, which were applicable to both strong and weak nonlinear equations, was reported^31^. For the entire solution domain, the derived approximate analytical solutions were valid. To make up for the disadvantages of conventional perturbation methods, a variety of modified perturbation approaches was proposed, along with some mathematical tools including variational theory, homotopy technology, and iteration technique. He's frequency formulation, the max–min approach, and the HPM were some of the simplest methods for nonlinear oscillators that were studied^32^. The He's frequency formulation was explained mathematically, and the weighted average was included to increase the predicted frequency accuracy. Strongly nonlinear oscillators were suggested to be approached in a straightforward and exclusive way^33^. The outcomes demonstrated that the approach provided a roughly accurate response. The survey offered a highly effective instrument for a quick approximation of the amplitude–frequency connection of a nonlinear oscillator, considering the simplest solution technique. A packaging system and a micro-electro-mechanical system under investigation both depend on the nonlinear relationship between the frequency and amplitude of a nonlinear vibration system^37^. A simple frequency prediction method for nonlinear oscillators with variable beginning conditions was put forth. The present paper developed a very practical method for quickly and precisely understanding a nonlinear vibratory system vibration property.

The PHI6 has prospective applications in a wide range of physical and technical phenomena when considering understanding in light of the advantages mentioned above. Furthermore, there are several applications in science and engineering for permanent employment investigations on the stability analysis of the parameterized PHI6. This equation represents a second-order ODE with a cubic-quintic nonlinearity. As a novel approach, we will use a non-perturbative strategy to produce a better estimate solution. It is compared to a numerical methodology to confirm the prototypical connection. The following details should be emphasized in relation to the adopted novel technique or significant results:

1. Simply expressed, the unique method generates a second equivalent linear differential equation that is the same as the existing nonlinear one.
2. An excellent match between these two equations is necessary for the new technique to work.
3. All conventional techniques use the Taylor expansion to lessen the complexity of the given problem when restoring forces are present. According to the current plan, this weakness is no longer present.
4. Unlike other conventional methodologies, the current methodology enables us to analyze the stability analysis of the issue.
5. The novel method appears to be a straightforward, practical, and fascinating tool. It can be used to analyze a variety of nonlinear oscillator classes.

To crystalize the implementation of our inquiry, the rest of the paper will be outlined along with the following sections: An improved estimate solution to the considered problem using a non-perturbative approach is introduced in section “Methodology of the presence of time-delay”. This section has tackled the two cases in the absence/presence of the time delay factor. In section "Discussion of the results", a discussion of the obtained results in view of some plots is presented. Finally, the primary outcomes of the current study are summarized in section “Conclusions”.

## Methodology of the presence of time-delay

The PHI6 and its excitation with positional delay were mostly covered in the following form:

6 $$\ddot{x} - \mu (1 - x(t - \tau )^{2} )\dot{x} + \omega^{2} x + \alpha x^{3} + \lambda x^{5} = F\cos \sigma t,$$

where all of the quantities employed for Eq. (1) have already been given.

It is desirable to imagine the preliminary situation as follows:

7 $$x(0) = A,\quad \dot{x}(0) = 0.$$

As stated in the original specifications of the oscillator as given in Eq. (6), one can establish the testing solution as follows:

8 $$u(t) = A\cos \omega t,\quad \dot{u}(t) = - A\omega \sin \omega t,$$

where *A* is the oscillation amplitude and $\omega$ is the natural frequency of the delayed Eq. (6).

It is required to evaluate the so-called equivalent frequency, which will be determined later. Additionally, an equivalent damping term $\zeta$ should be evaluated.

Suitably, the shift delayed response $x(t - \tau )$ has the subsequent development:

9 $$\begin{aligned} u(t - \tau ) & = A\cos \omega (t - \tau ) \\ & = A(\cos \omega t\cos \omega \tau + \sin \omega t\sin \omega \tau ) \\ & = u(t)\cos \omega \tau - \frac{1}{\omega }\dot{u}(t)\sin \omega \tau . \\ \end{aligned}$$

The estimate frequency is best derived through the use of weighted residual technology. The frequency of the generalization of odd terms in the controlling equation of motion can be calculated by using He's formalism. The frequency can be roughly calculated by using the weighted residuals by Ren^38^ approach. Applying his methodology and Eqs. (6), (8), and (9), one concludes that

10 $$\Omega^{2} = \int\limits_{0}^{2\pi /\omega } {u\left( {\omega^{2} u + \alpha u^{3} + \lambda u^{5} } \right)dt/\int\limits_{0}^{2\pi /\omega } {u^{2} dt} } .$$

The calculations show that

11 $$\Omega^{2} = \frac{5}{8}\lambda A^{4} + \frac{3}{4}\alpha A^{2} + \omega^{2} .$$

As shown from Eq. (11), the equivalent frequency depends on all physical parameters except the damping parameter *μ*. Now, following El-Dib^39, 40^, the equivalent damping parameter may be formulated by the following rule:

12 $$\zeta = \int\limits_{0}^{2\pi /\omega } {\dot{u}\left( {1 - (u\cos \omega \tau - \dot{u}\sin \omega \tau /\omega )^{2} } \right)} dt/\int\limits_{0}^{2\pi /2} {\dot{u}^{2} dt} .$$

Employing Eqs. (8) and (9) in Eq. (12), then using the Mathematica Software 12.0.0.0, one achieves

13 $$\zeta = - \frac{\mu }{4}(4 - 2A^{2} + A^{2} \cos 2\omega \tau ).$$

It should be noted that Eq. (13) is a coupling equation with the equivalent frequency (11). Therefore, a transcendental equation with (11) is obtained. Finally, the equivalent linear ODE to the PHI6 may be written in the following form:

14 $$\ddot{u}(t) + \xi \dot{u}(t) + \Omega^{2} u(t) = F\cos \sigma t.$$

On the other side, in the absence of the excited force, one can deduce that stability criteria. Equation (14) then become:

15 $$\ddot{u}(t) + \zeta \dot{u}(t) + \Omega^{2} u(t) = 0.$$

The normal form of Eq. (15) can be achieved along with the substitution $u(t) = f(t)Exp( - \zeta t/2)$. In this case, Eq. (15) will be transformed into the subsequent equation:

16 $$\ddot{f}(t) + \Lambda^{2} f(t) = 0,$$

where $$\Lambda^{2} = \Omega^{2} - \zeta^{2} /4$$.

So, the stability criteria occur provided that:

17 $$\Lambda^{2} > 0,\quad {\text{and}}\quad \zeta > 0.$$

For more convenience, one can plot $\Lambda^{2} \;\;{\text{and}}\;\;\zeta$ as functions in the natural frequency $\omega$.

On the reverse side, Eq. (14) is a linear ODE, and its solution is directly obtained. According to this equation, one can predict that this solution has a positive impact on the different values of the damping parameters $A,\;\mu ,$ and the natural frequency $\omega$, therefore the solution depends directly on these parameters. On the same procedure, values of the potential parameters $\alpha$ and $\lambda$ have no influence on the behavior of the solution, while the variation of $\sigma$ is expected to be slight.

Now, going back to the fundamental time-delay Eq. (6), the non-perturbative methodology allows us to construct the same initial conditions (7) which correspond to the linear equation, as shown in Eq. (14). As previously said, all the parameters occur in the equivalent frequency as shown in Eq. (11).

It is convenient to compare the analogy between the linear ODE solution (non-perturbative solution) and the numerical solution of the original Eq. (6) using the Mathematica Software 12.0.0.0. Therefore, the following values of the used parameters are considered.

$$\mu = - 0.5,\;\;\sigma = 1.5,\;\;\alpha = 1.5,\;\;F = 0.02,\;\;A = 0.5,\;\;\tau = 0.1,\;\;\omega = 4.0,\;\;\lambda = 0.05.$$

**Figure 1:**
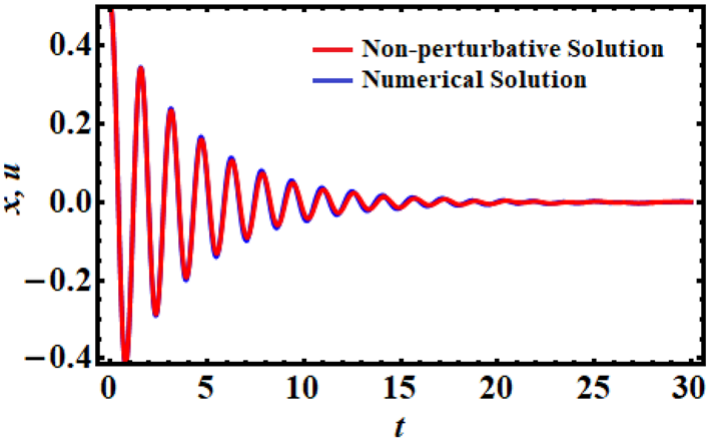
Describes an excellent matching between the non-perturbative solution of Eq. (14) with the numerical solution of Eq. (6) at $\tau = 0.1$.

The comparison demonstrates that the two solutions have a great constancy with each other, as illustrated in Fig. 1, where both solutions are represented in the same figure. Additionally, the Mathematica Software demonstrated that the absolute error between the analytical and numerical solutions is 0.0173 up to a time of 30 units.

### Approach of the absence of time-delay

The PHI6 and its position delay excitement were primarily addressed in the following way:

18 $$\ddot{x} - \mu (1 - x^{2} )\dot{x} + \omega_{0}^{2} x + \alpha x^{3} + \lambda x^{5} = F\cos \omega t.$$

It is preferable to picture the first circumstance as previously shown in Eq. (7).

**Figure 2:**
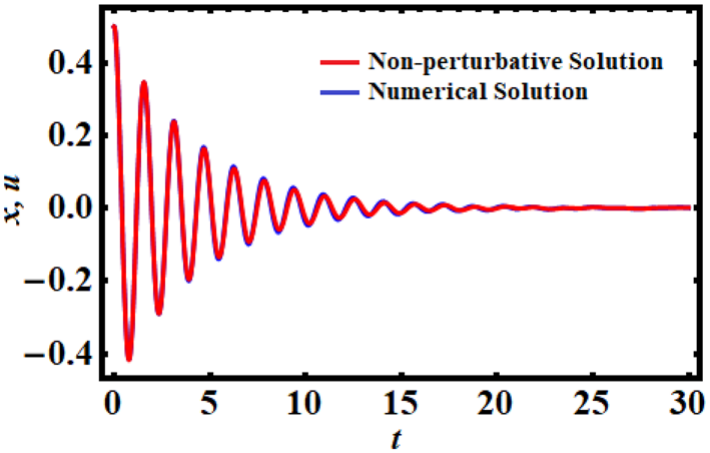
A matching between the original Eq. (6) and the linear Eq. (14) in the absence of time-delay.

Following the related arguments as in the previous Section, simply setting $\tau = 0$, one gets the same equivalent frequency, meanwhile, the equivalent damping term differs from the previous methodology by canceling the time-delay factor. Therefore, Fig. 2 is plotted for the same previous data as shown in Fig. 1, but in the absence of the time-delay factor. The Mathematica Software 12.0.0.0 also showed that, up to a time interval of 30 units, the absolute error between the analytical and numerical solutions is 0.0182106.

## Discussion of the results

**Figure 3:**
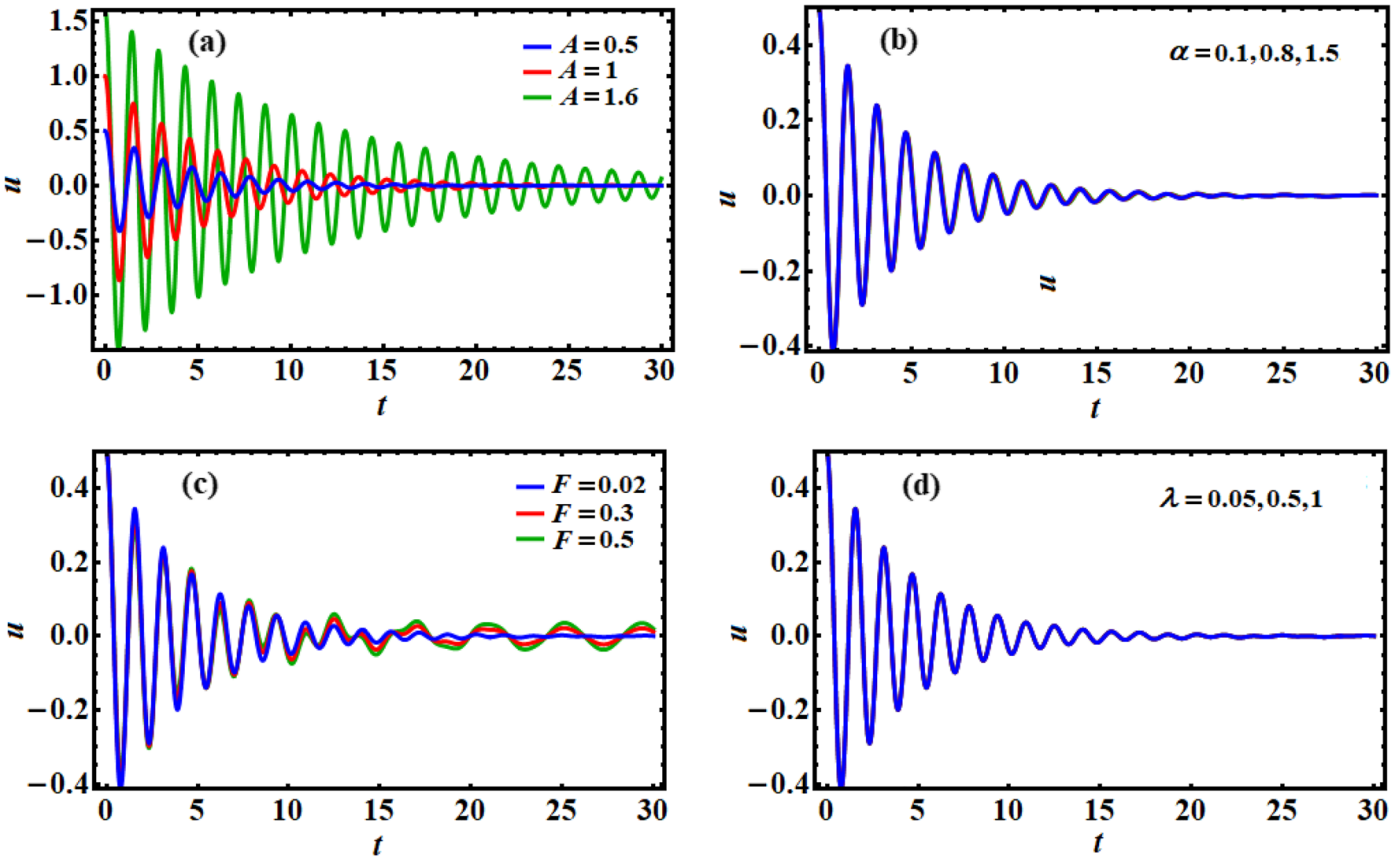
Explores the time history of the solution $u(t)$ of Eq. (14) for distinct values of: (**a**) $A$, (**b**) $\alpha$, (**c**) $F$, and (**d**) $\lambda$.

**Figure 4:**
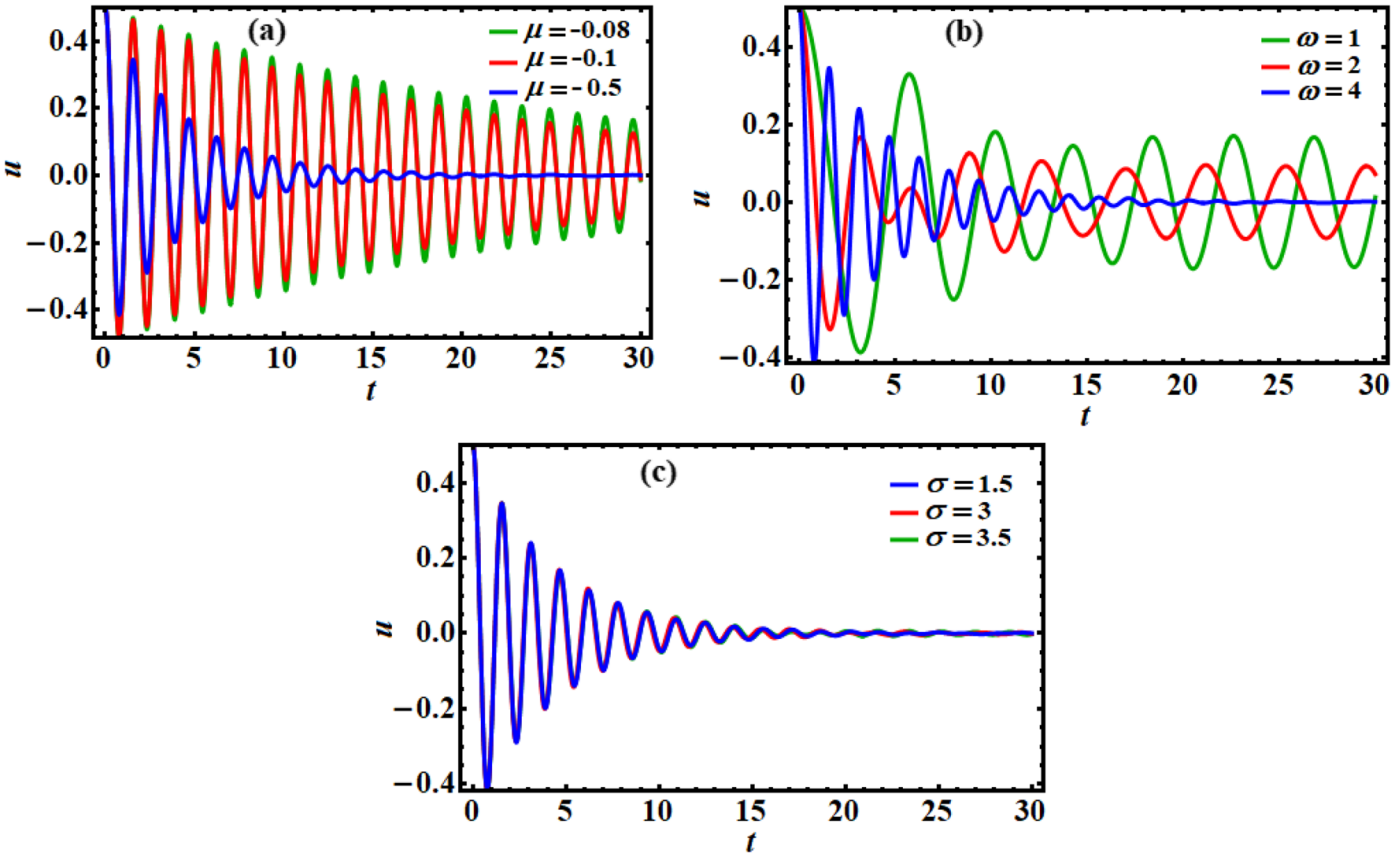
Shows the behavior of $u(t)$ for various values of: (**a**) $\mu$, (**b**) $\omega$, (**c**) $\sigma$.

This section is demonstrated to shed light on the obtained outcomes in view of the aforementioned data. Therefore, Figs. 3 and 4 are drawn to describe the temporal behavior of $u(t)$ at various values of $A,\;\alpha ,\;F,\;\lambda ,\;\mu ,\;\omega ,$ and $\sigma$. An inspection of the drawn curves in Fig. 3a shows that the function $u(t)$ has decay behavior during the investigated period of time, which means that the solution goes to a stable behavior through the whole interval [0,30]. As predicted before, the increase of *A* produces an increase in the wave numbers and amplitudes. Moreover, the solution reaches the stability behavior faster with the decrease of these values. The variation of *u*(*t*) according to *F* values is noted in part (c) of Fig. 3, in which it becomes clearly visible in the last two-thirds of the time period. On the other side, it is observed that there is no change in the solution with the change in the values of $\alpha$ and $\lambda$, as seen in parts (b) and (d) of Fig. 3. The reason goes back to the mathematical formula of Eq. (14).

As expected before, the solution of Eq. (14) varies with the change of *μ* and $\omega$, while the resulting variation with the values of $\sigma$ seems slight, as seen in parts of Fig. 4. This solution reaches faster to the stability manner with the increase of *μ* and $\omega$, as indicated in the first two parts of Fig. 4, respectively.

**Figure 5:**
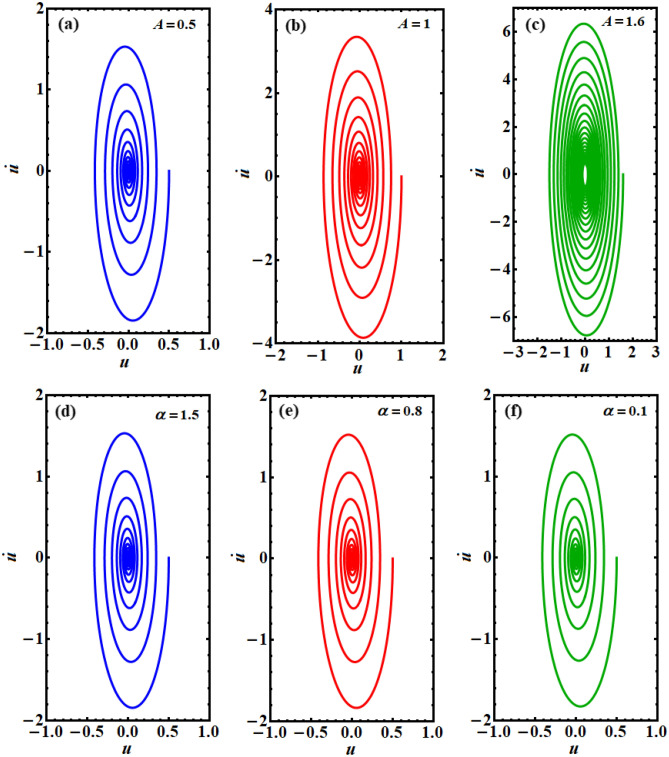
Shows the phase plane plots of the solution $u(t)$ at: (**a**) $A = 0.5$, (**b**) $A = 1$, (**c**) $A = 1.6$, (**d**) $\alpha = 1.5$, (**e**) $\alpha = 0.8$, (**f**) $\alpha = 0.1$.

**Figure 6:**
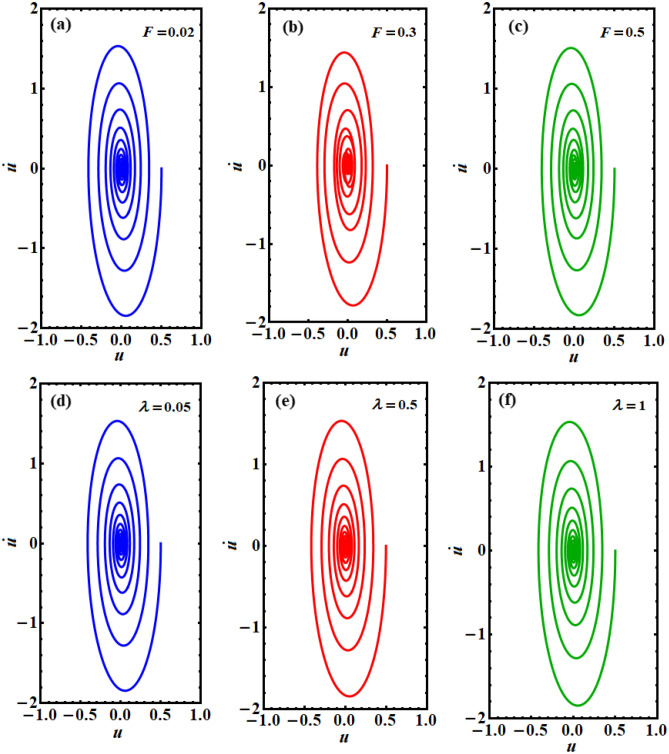
Represents the phase plane plots at: (**a**) $F = 0.02$, (**b**) $F = 0.3$, (**c**) $F = 0.5$, (**d**) $\lambda = 0.05$, (**e**) $\lambda = 0.5$, (**f**) $\lambda = 1$.

**Figure 7:**
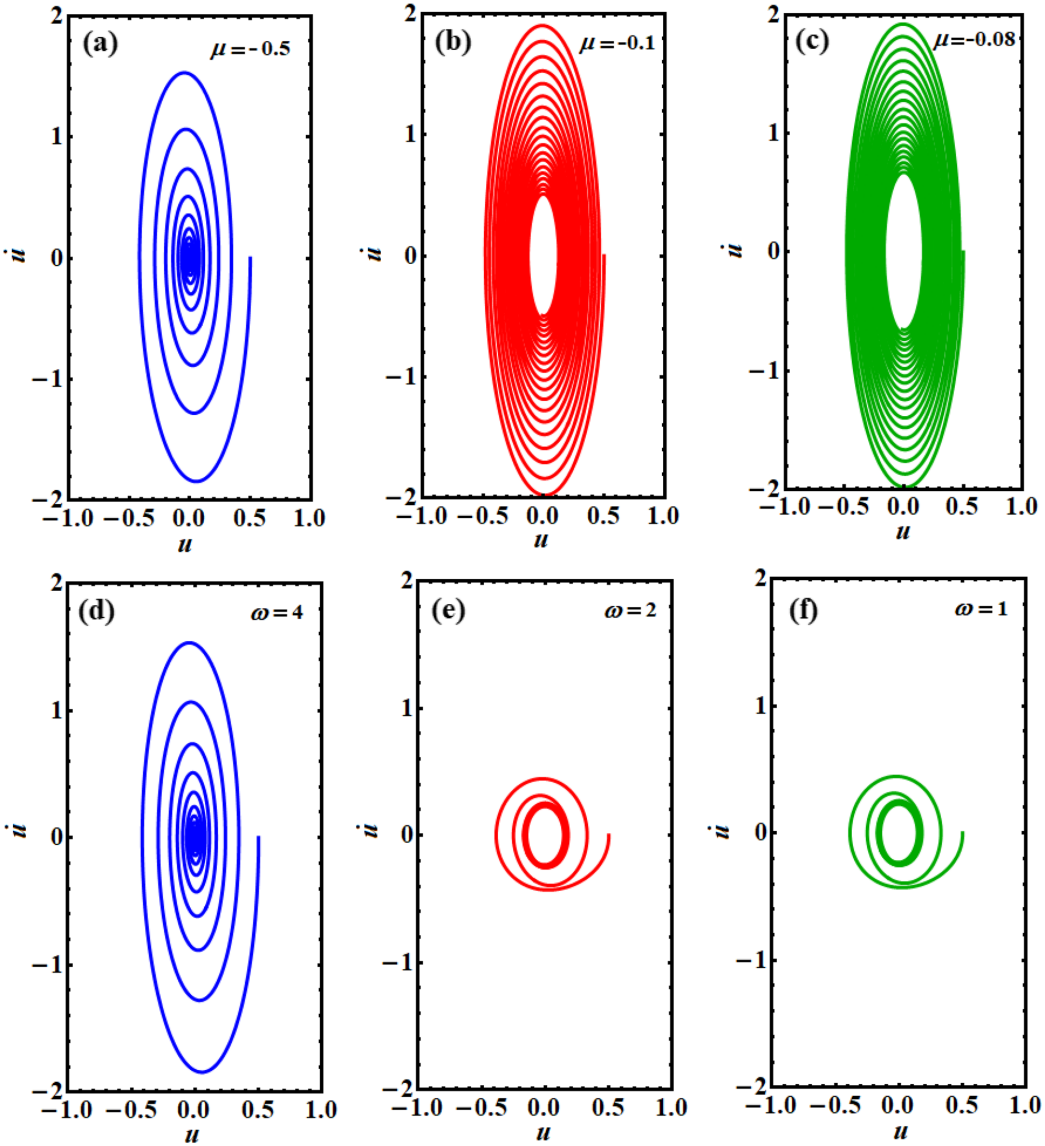
Reveals the curves in the plane $(u - \dot{u})$ at: (**a**) $\mu = - 0.5$, (**b**) $\mu = - 0.1$, (**c**) $\mu = - 0.08$, (**d**) $\omega = 4$, (**e**) $\omega = 2$, (**f**) $\omega = 1$.

The relation between the solution *u* and its first derivative $\dot{u}$ is plotted in the plane $(u - \dot{u})$ to reveal the phase plane diagrams of this solution, as drawn in Figs. 5, 6, 7. One can observe that all of the drawn curves have spiral forms towards inside one point, which asserts the stability of the obtained solution. It is found that these curves completely agree with the effect of the parameters used above in the Figs. 3 and 4. Additionally, it is noted that the parameters $A,\;F,\;\mu ,$ and $\omega$ have more positive impact on the phase plane curves than the others, as seen in the various portions of Figs. 5, 6, 7. These curves reflect the time behavior of the represented solution in parts of Figs. 3 and 4. In other words, the phase plane plots in Fig. 5 correspond to the same behavior of the time history curves in Fig. 3a,b, while curves in the plane $(u - \dot{u})$, as in Figs. 6 and 7 express the plotted waves in Figs. 3a,d and 4a,b, respectively. Based on the good impact of the various values of the parameters $A,\;F,$ and $\mu ,\;\omega$ on the solution’s waves, as in Figs. 3a,c and 4a,b, one can see the positive variation of the phase plane diagrams, as explored in Figs. 5a–c, 6a–c, and 7a–c, d–f, respectively. On the other hand, there is no observed influence on the curves of phase plane diagrams when $\alpha$ and $\lambda$ have different values, as seen in portions (d), (e), (f) of Figs. 5 and 6, respectively. This behavior corresponds to the related portions in Fig. 3.

**Figure 8:**
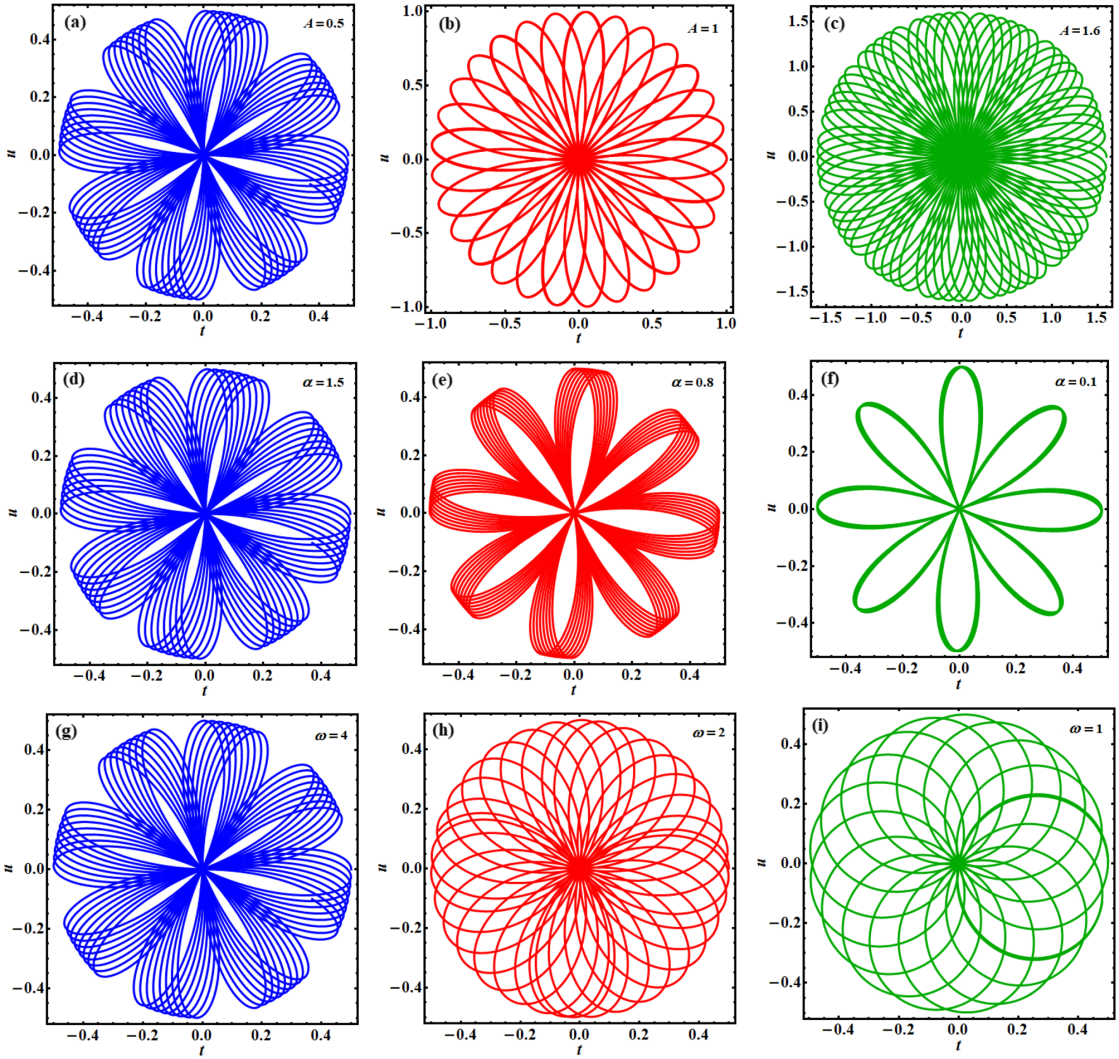
Shows the polar forms of $u(t)$ for various values of: (**a**–**c**) $A$, (**d**–**f**) $\alpha$, (**g**–**i**) $\omega$.

Moreover, several polar forms of Eq. (8) have been plotted in parts of Fig. 8 in light of the values of the parameters $A,\;\alpha ,$ and $\omega$. The inspection of these parts demonstrates that the values of the amplitude, potential, and the frequency have a direct bearing on the periodicity of the solution obtained. It is remarked that as the value of A grows, more waves are depicted by symmetric elliptical curves that cross in the middle of each portion, as seen in portions (a)–(c) of Fig. 8. It is found that these curves decrease significantly with the decrease in the potential value, displayed in portions (d)–(f) of the same figure. As for the good effect of the frequency values, we find that the parts (g)–(i) of Fig. 8 express that the density of the grid, that forms the drawn curves, increases with the increase of the frequency value, and reaches its symmetrical form at the largest value of the frequency.

**Figure 9:**
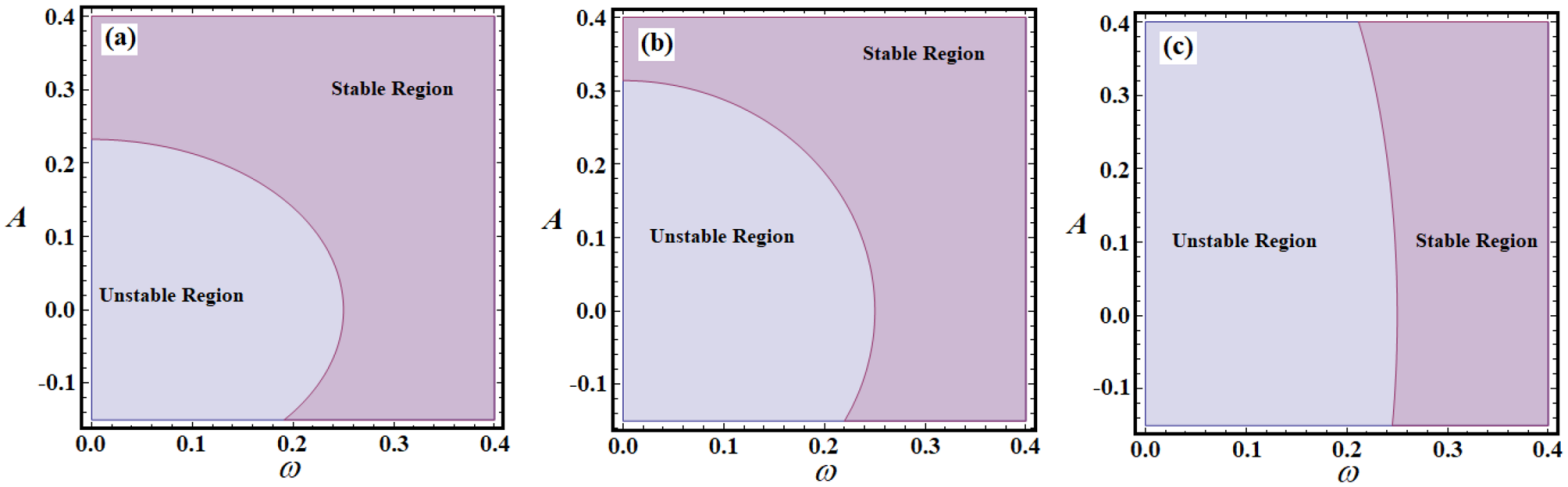
Shows the stability and unstable regions of relations (17) for various values of $\alpha$: (**a**) $\alpha = 1.5$, (**b**) $\alpha = 0.8$, (**c**) $\alpha = 0.1$.

**Figure 10:**
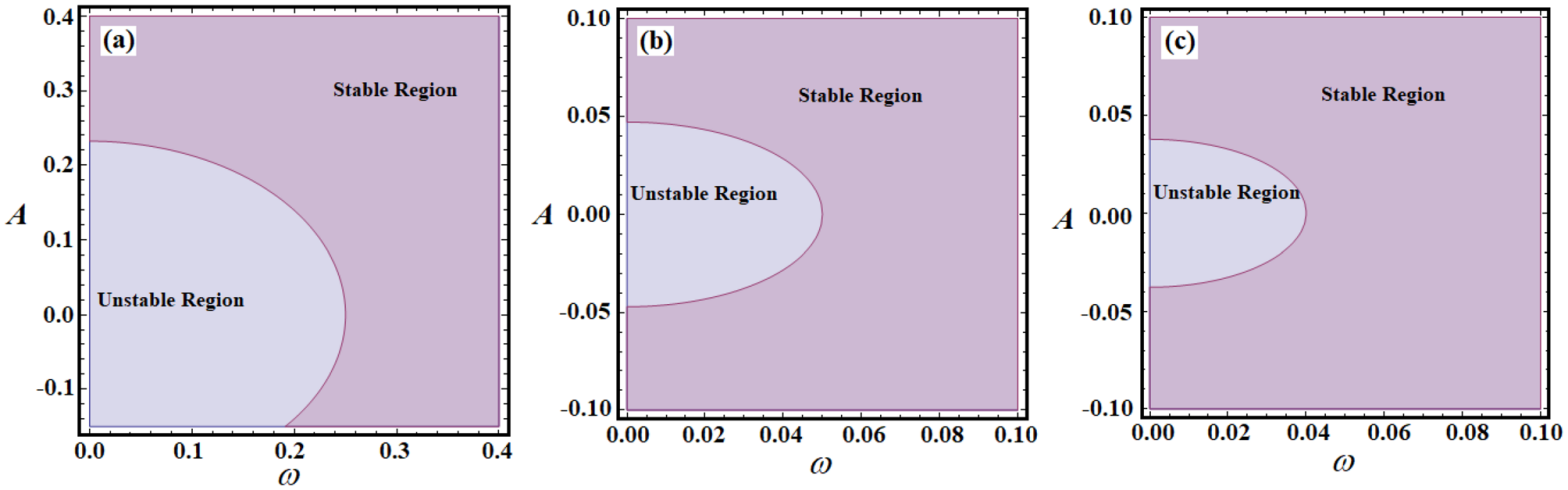
Reveals the zones of stability and unstable according to relations (17) for various values of $\mu$: (**a**) $\mu = - \;0.5$, (**b**) $\mu = - \;0.1$, (**c**) $\mu = - \;0.08$.

**Figure 11:**
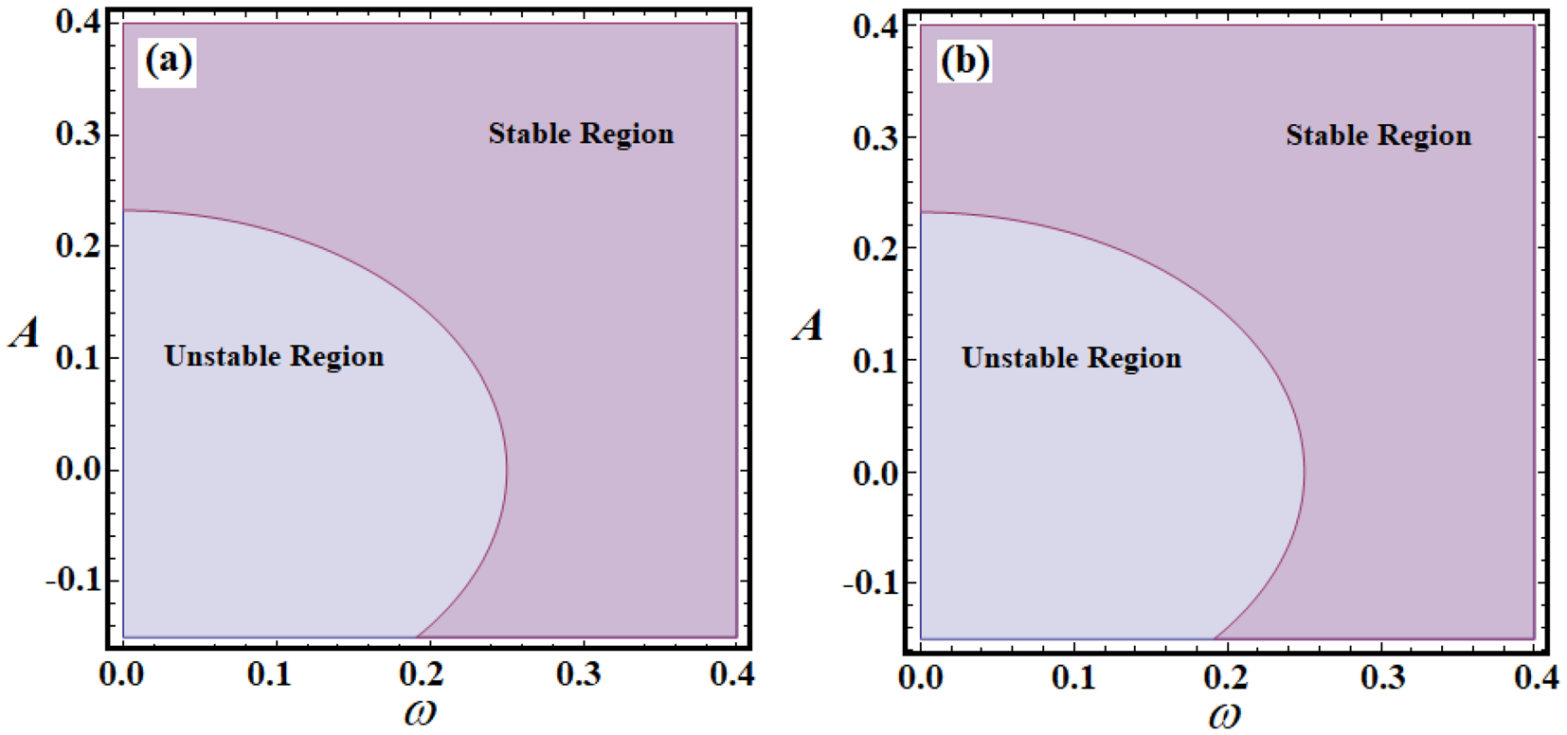
Sketches the stability and unstable areas for different values of: (**a**) $\lambda ( = 0.05,\;0.5,\;1)$, (**b**) $\sigma ( = 1.5,\;3,\;3.5)$.

The stable and unstable ranges of relations (17) according to values of $\alpha ,\;\mu ,\;\sigma ,$ and $\lambda$ are depicted in parts of Figs. 9, 10, 11. The drawn curves in this figure are calculated when $\alpha ( = 0.1,\;0.8,\;1.5)$, $\mu ( = - 0.5,\; - 0.1,\; - 0.08),$ $\sigma ( = 1.5,\;3,\;3.5),$ and $\lambda ( = 0.05,\;0.5,\;1)$, as in Figs. 9a–c, 10a–c, and 11a,b. These figures depict the fluctuation of *A* versus $\omega$. Observing the plotted regions in these potions reveals that as the values of $\alpha$ decrease, the stability zone shrinks, while the instability region expands, as seen in portions (a), (b), and (c) of Fig. 9. The reason goes back to the first condition in (17), in which the decrease of $\alpha$ values produces decrease of the $\Omega^{2}$ values, and then these zones will be influenced by this change. On the contrary, with the variation of *μ*, i.e., when the values of *μ* increase, the areas of stability increase, as drawn in Fig. 10a–c. The reason is due to the fact that the chances of $\Omega^{2} > \zeta^{2}$ are increased by increasing the values of $\zeta$. Moreover, it is noted that there is no variation with the change of the parameters $\lambda$ and $\sigma$. The reason is based on that $\lambda$ has no effect on the value of $\Omega^{2}$ explicitly, in addition the conditions (17) are obtained in the absence of external forces. In other words, the stability and instability areas remain stationary. The reason for the increase in the region of stability is due to the mathematical configuration of relations (17).

## Conclusions

Numerous physical, electrical, industrial, and technical machines are displayed along with the Duffing oscillator prototype. The Van der Pol oscillator, on the other hand, is a model for frequently self-excited oscillators seen in physics, engineering, semiconductors, physiology, neurophysiology, and many other disciplines. As a conclusion, the purpose of the current study is to couple these two equations to create the PHI6 prototype. A time-delay in the square position has been employed in the current work to examine the stability analysis. This study is essential since time-delayed technology has recently been the subject of several inquiries. A novel non-perturbative method is applied to obtain an equivalent frequency, damping term, and ultimately an equivalent linear differential equation. It has become apparent that every physical variable in the basic governing equation of motion is included in the comparable frequency/damping terms. To confirm the accuracy of the theoretical results, verification with a numerical approach is recommended. The examination has been produced by using the analytical technique of practical approximation. In contrast to earlier investigations, the novel approach arriving at the current solution is significant for being effective, prospective, and simple. It is determined whether the multiple scale method may be applied to further nonlinear oscillators using the organized nonlinear prototype approach. Therefore, to handle the multiple time scale of the considered PHI6, it is easier to take advantage of the equivalent linear ODE. It has studied how different regulatory constraints affected the foundation vibration accomplishments. The following key points summarize the results of the current attempt:

- Instead of the PHI6 prototype, an equivalent linear ODE is given in Eq. (14).
- A good matching between the two equations is displayed throughout Figs. 1 and 2 for the cases of presence/absence on the time delay, respectively.
- The phase plane diagrams of the equivalent linear ODE are displayed across Figs. 5, 6, 7.
- Several polar forms of the resulted solution are displayed to express that the grid density, which creates the sketched curves, rises with the rise of the frequency values, and takes on its symmetrical form at the highest frequency.
- The stability charts are depicted in Figs. 9, 10, 11.

In regard to the implemented distinctive technique or noteworthy outcomes, the following information should be highlighted:

1. The novel method produces a second equivalent linear differential equation that is identical to the current nonlinear one, to put it simply.
2. For the new method to operate, these two equations must perfectly match one another.
3. When restoring forces are present, all conventional methods apply the Taylor expansion to reduce the difficulty of the given problem. This weakness no longer exists under the current plan.
4. Unlike other traditional approaches, the one we use now allows us to examine the issue's stability analysis.
5. The unique approach seems to be a simple, useful, and intriguing tool. It is applicable to the analysis of numerous nonlinear oscillator categories.

6. The achieved results generalize those obtained in^2–6^, in the absence of time delay.

Therefore, as a future work, the previous non-perturbative analysis will be adopted to depict several classes in the dynamical systems. Additionally, the complicated topics of nonlinear dynamics with complex coefficients can also be analyzed.

## Data Availability

All data generated or analysed during this study are included in this published article.
